# Determination of Diclofenac on a Dysprosium Nanowire- Modified Carbon Paste Electrode Accomplished in a Flow Injection System by Advanced Filtering

**DOI:** 10.3390/s91007903

**Published:** 2009-09-30

**Authors:** Parandis Daneshgar, Parviz Norouzi, Mohammad Reza Ganjali, Rasoul Dinarvand, Ali Akbar Moosavi-Movahedi

**Affiliations:** 1 Center of Excellence in Electrochemistry, Department of Chemistry, University of Tehran, Tehran, Iran; E-Mails: daneshgar@khayam.ut.ac.ir (P.D.); ganjali@khayam.ut.ac.ir (M.R.G.); 2 Endocrinology & Metabolism Research Center, Tehran University of Medical Sciences, Tehran, Iran; 3 Medical Nanotechnology Research Center, Faculty of Pharmacy, University of Tehran Medicinal Sciences, Tehran, Iran; E-Mail: dinarvand@sina.tums.ac.ir (R.D.); 4 Institute of Biochemistry and Biophysics, University of Tehran, Tehran, Iran; E-Mail: Moosavi@ibb.ut.ac.ir (A.A.M.-M.)

**Keywords:** Fast Fourier Transformation, square wave voltammetry, carbon paste electrode dysprosium nanowire, diclofenac, oxidation-reduction

## Abstract

A new detection technique called Fast Fourier Transform Square-Wave Voltammetry (FFT SWV) is based on measurements of electrode admittance as a function of potential. The response of the detector (microelectrode), which is generated by a redox processes, is fast, which makes the method suitable for most applications involving flowing electrolytes. The carbon paste electrode was modified by nanostructures to improve sensitivity. Synthesized dysprosium nanowires provide a more effective nanotube-like surface [1-4] so they are good candidates for use as a modifier for electrochemical reactions. The redox properties of diclofenac were used for its determination in human serum and urine samples. The support electrolyte that provided a more defined and intense peak current for diclofenac determination was a 0.05 mol L^−1^ acetate buffer pH = 4.0. The drug presented an irreversible oxidation peak at 850 mV vs. Ag/AgCl on a modified nanowire carbon paste electrode which produced high current and reduced the oxidation potential by about 100 mV. Furthermore, the signal-to-noise ratio was significantly increased by application of a discrete Fast Fourier Transform (FFT) method, background subtraction and two-dimensional integration of the electrode response over a selected potential range and time window. To obtain the much sensivity the effective parameters such as frequency, amplitude and pH was optimized. As a result, C_DL_ of 2.0 × 10^−9^ M and an LOQ of 5.0 × 10^−9^ M were found for the determination for diclofenac. A good recovery was obtained for assay spiked urine samples and a good quantification of diclofenac was achieved in a commercial formulation.

## 1. Introduction

Diclofenac sodium (DFNa) (sodium [*o*-(2,6-dichloroanilino)phenyl] acetate), is a non-steroidal anti-inflammatory drug (NSAID) with strong anti-pyretic, analgesic and anti-inflammatory properties [1]. It is widely used in clinical medicine for the treatment of inflammatory conditions such as rheumatoid arthritis, osteoarthritis and ankylosing spondilytis [2,3]. The efficacy of diclofenac equals that of many newer and established NSAIDs. As an analgesic, it has a fast onset and a long duration of action. Compared to other NSAIDs, diclofenac is well tolerated and rarely produces gastrointestinal ulcerations or other serious side effects. Thus, diclofenac can be considered as one of few non-steroidal anti-inflammatory drugs of first choice used in the treatment of acute and chronic, painful and inflammatory conditions [4]. To date, several methods for determination of diclofenac have been reported. These include potentiometry [5-7], capillary zone electrophoresis (CZE) [8], high performance liquid chromatography (HPLC) [9-11], high-performance liquid chromatography-mass spectrometry (HPLC-MS) [12], spectrophotometric [13], spectrofluorometric [14-16], thin layer chromatography [17], gas chromatography [18], polarographic analysis [19], spectrophotometric [20-24] and chemometric techniques [25-27]. To the best of our knowledge, very little has been reported on voltammetric determination of diclofenac sodium, because the electrooxidation of diclofenac sodium proceeds very slowly and almost no current response was observed at the usual electrode.

The method introduced in this paper for detection of diclofenac is very sensitive, inexpensive and fast. Squuare Wave Voltammetry (SWV) has recently been shown to be advantageous for environmental detection of several compounds [28]. The adaptation of this technology to SWV of diclofenac on a Dysprosium nanowire/carbon paste electrode(DyNW/CPE) could provide a substantial improvement for rapid and very sensitive analysis [29,30].

Carbon-paste electrodes (CPEs), due to their ease of construction, renewability, and compatibility with various types of modifiers, have been widely used as a suitable matrix for preparation of modified electrodes. Further, they show rather low background current compared to solid graphite or noble metal electrodes [31]. In recent years, applications of carbon nanotube (CNT) modified carbon paste electrodes have provided considerable improvements in the electrochemical behavior of biologically important compounds [32,33]. Metal nanowires such as dysprosium showed behavior like CNTs. A CPE containing 10% (w/w) of DyNW, in comparison with CPE without nanowire, showed a very effective catalytic activity in the electrochemical oxidation of diclofenac.

Use of the Fast Fourier Transform method was found to provide a very sensitive system in combination with an electrochemical method for trace detection of several compounds [34-41]. This paper describes a fundamentally different approach to SWV measurement, in which the detection limits are improved, while preserving the information content of the SW voltammogram. The approach is designed to separate the voltammetric signal and background signal in the frequency domain by using a discrete Fast Fourier Transformation (FFT) method. SWV measures the current response while rapid alternating potentials are applied during a staircase scan, whereas CV, which uses only a forward and reverse linear dc scan, is not sensitive to the potential dependence of changes that occur in the double layer. However, the reported methods suffer from limitations such as material waste and long analysis times, because a number of preliminary steps are often required to obtain the species from the sample matrix. In this paper a very simple and sensitive electrochemical method for determination of diclofenac was introduced.

## 2. Experimental Section

### 2.1. Instrumentation

An electrochemical instrument for ultra voltammetry, and a homemade potentiostat were used for the reported voltammetric measurements. All electrochemical experiments were done using a setup comprised of a Pentium IV PC equipped with a data acquisition board (PCL-818H, Advantech Co.) that was used to output an analog waveform to the working electrode and acquire current readings from the working electrode that was connected to a custom made potentiostat. The card and accompanying dynamic link libraries allowed waveform generation and current sampling to be synchronized, which was essential in interpreting SWV current response. The memory and CPU requirements of the computer were dictated by the nature of the data acquisition requirements. Software was developed using Delphi 6.0 to repeatedly apply a waveform to the working electrode and synchronously acquire, analyze, and store the current data. The data could be interpreted in real time, or stored data could be loaded and reanalyzed to generate electropherograms. The algorithms used to interpret the current response from each waveform cycle were previous reported in [42]. Most of the waveform parameters could be modified from within the software; including the pre- and post scan potential/time, square wave frequency/amplitude, dc ramp initial/final potential, and ramp time.

### 2.2. Carbon paste electrode

**Figure 2.**
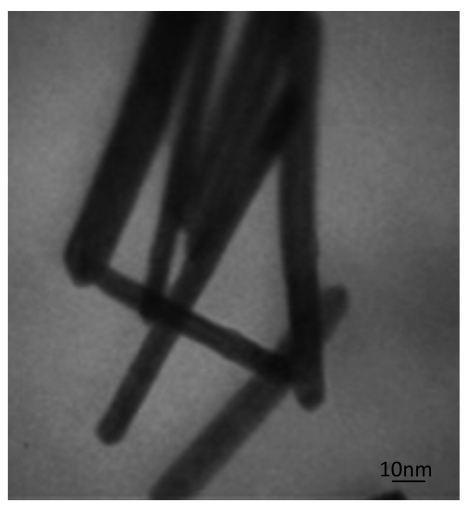
TEM image of dysprosium nanowire.

The nanowires was synthesized based on the procedure described by Li *et al.* [43,44]. A TEM image of a dysprosium nanowire was presented in Figure 2. The dysprosium nanowire carbon paste electrode (DyNW/CPE) was prepared by hand-mixing 0.97 g of graphite powder, 0.03 g of DyNW, and 0.34 mL paraffin oil adequately in an agate mortar. A portion of the resulting paste was then packed firmly into the electrode cavity (1.0 mm diameter) of a polytetrafluorethylene (PTFE) sleeve. The unmodified CPE was prepared in a similar way using 1.25 g of graphite powder and 0.45 mL of paraffin oil. Electrical contact was established via a copper wire. The surfaces of all the modified and unmodified CPEs were carefully smoothed on weighing paper and rinsed with twice distilled water prior to each measurement.

### 2.3. Materials and reagents

All chemicals and reagents were of analytical grade quality. All the solutions was made with double distilled water. Diclofenac was a gift from Drug and Food Quality Control (Tehran, Iran). A stock solution of 1.0 × 10^−5^ M of diclofenac was prepared at 4 °C. More dilute solutions were prepared daily with deionised water just before use. The phosphate buffer (pH 3–9), Tris-HCl and acetate buffer were prepared using analytical grade reagents and were used as supporting electrolytes.

### 2.4. Stripping voltammetry

**Figure 3.**
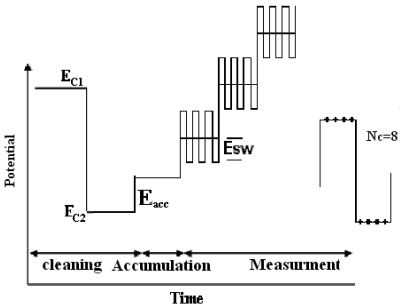
Diagram of the potential waveform used in measurements.

In this new method to improve the detector sensitivity, the potential excitation waveform and current sampling and data processing of the FFT-SWV technique were modified (Figure 3). The potential waveform consisted of three sections; (a) electrode conditioning, (b) accumulation part and (c) measurement. The potential waveform contained three additional potential steps: E_c1_ to E_c2_ (for cleaning the electrode surface) and Es (for accumulation of diclofenac). As is shown in Figure 2, the measurement part of the waveform contains multiple SW pulses with amplitude of *E*_sw_ and frequency of *f*_o_, that were superimposed on a staircase potential function, which was changed by a small potential step of ΔE. The values of potential pulse of SW (E_SW_) and ΔE were in a range of few mV (10 to 50 mV). In the potential ramp, the currents were sampled four times per each SW polarization cycle. After preparing the solution, the measurements were carried out in the continuous Fast Fourier Transform Stripping Square Wave Voltammetric (FFTSW) mode. A typical experiment consisted of three consecutive steps with the following experimental conditions: the pre-concentration at 0.1 V versus Ag/AgCl for 20 s, and a polarization (stripping step) run from 0.6 V to 1.0 V by applying a ƒ = 600 Hz, and the pulse height, Esw = 50 mV. The flow rate was set at 0.5 mL/min. it is optimized flow rate as mentioned in our previous papers [45,46] by considering the best Signal to Noise ratio (S/N) for the electroanalytical signal of injection of 1.0 × 10^−7^ M of a component such as Fe^3+^.

### 2.5. Sample preparation assay

Twenty tablets were weighed, finely powdered and portions equivalent to 100 mg diclofenac were transferred into a 250 mL volumetric flask; 100 mL of distilled water was added, shaken thoroughly to dissolve, made up to volume and mixed well. Suitable aliquots of solution were filtered through a Millipore filter (0.45 μm). One mL of the filtered solution was diluted with distilled water in a 100 mL volumetric flask. Then 50 μL of the resulting solution was added to a 100 mL volumetric flask and made up to volume with 0.05 M acetate buffer to yield a starting concentration of 0.06 μM.

### 2.6. Determination of diclofenac in human urine and plasma

One mL of untreated urine containing 500 ng/mL diclofenac was placed in a 10 mL volumetric flask and diluted with water to the mark. A 1 mL aliquot of this solution was diluted with pH 4 buffer solution to 10 mL into a volumetric flask. Then a 50 μL aliquot was injected into the system. For the determination of diclofenac in plasma, 100 μL of aqueous diclofenac solutions (10 ng/mL) were added to 100 μL of untreated plasma. The mixture was vortexed for 30 s. In order to precipitate the plasma proteins, the plasma samples were treated with 50 μL perchloric acid HClO_4_ 15%. After that, the mixture was vortexed for a further 30 s and then centrifuged at 6,000 rpm for 5 min. Then a 50 μL aliquot of the obtained supernatant was injected into the system. The voltammograms were recorded according to the above recommended procedure. The voltammograms of samples without diclofenac do not show any signal that can interfere with the direct determination, so external calibration can be used.

## 3. Results and Discussion

### 3.1. Electrochemical behavior of diclofenac by using modified DyNW/CPE

**Figure 4.**
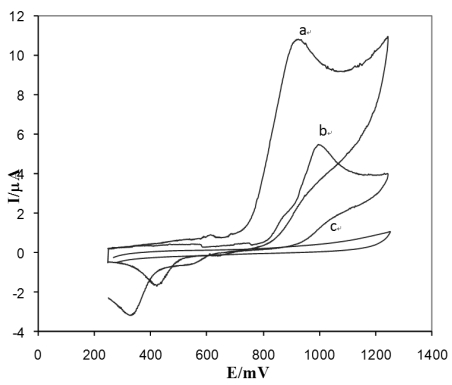
The voltammograms of 5.0 × 10^−7^ M diclofenac on the modified (a) and unmodified (b) carbon paste electrode at acetate buffer pH 4, (c) acetate buffer using NWCPE.

Figure 4a,b shows a relatively broad and weak anodic wave and one reduction peak for the electro-oxidation of diclofenac on the surface of the unmodified electrode that revealed that the electrode process is sluggish and it oxidized at a higher potential of about 950 mV.

On the other hand, using the DyNW-modified electrode, a well-defined and very sharp anodic wave with a peak potential of 850 mV is obtained for diclofenac. On the basis of these observations, it can be postulated that the addition of DyNW to the matrix of carbon nanotube modified paste electrode (CNTPE) works in an effective catalytic fashion in the electrochemical oxidation of diclofenac, leading to the remarkable enhancement of the anodic peak current. For diclofenac, in the potential range of 0–1.2 V (versus Ag/AgCl) there is an oxidation peak at 950 mV in the NWCPE which by increasing the potential scan rates, increases peak current. The anodic peak current at the DyNW/CPE is greatly enhanced (by approximately 80%) and this is accompanied by a negative shift of 80 mV in the oxidation potential. This indicates that the DyNW/CPE improves electrochemical reactivity toward the oxidation of diclofenac compared with a bare CPE.

### 3.2. Effect of pH

**Figure 5.**
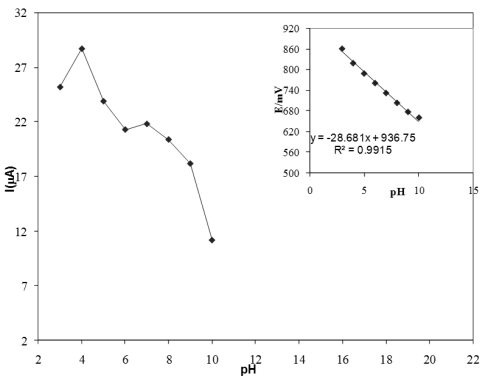
Influence of pH on the (a) peak current and (b)peak potential for 5.0 × 10^−7^ M in acetate buffer.

The influence of the Tris-HCl (pH 2.0–10), phosphate buffer (pH 2.0–10) and acetate buffer (pH 2.0–10) solution on the peak current was also checked after pre-concentration for 20 s at 0.2 V. The peak current was pH-dependent and much more developed in acetate buffer solution. Figures 5a,b present the dependence of the potential and current of the diclofenac oxidation on the solution pH at concentration of 5.0 × 10^−7^ M in acetate buffer. From the Ip on pH dependence: (a) it is seen that there are different ionic species, having different diffusion coefficients. As can be seen at Figure 4a, Ip increased from pH 2 to 4 and then decreased from pH 4 to 10. pH 4 was chosen because of the higher current providing more sensitivity for the determination. Furthermore, from Figure 4b it was found that the peak potential was shifted to more negative values with increasing pH 2.0–10. This indicated the presence of chemical reactions (proton-transfer reactions) in the electrode process. The following equation describes the correlation between peak potential and pH:E(mV) = −28.68 pH + 936.75

The slope was close to that expected for a two electron/one proton reaction, which was about 0.03 V at 25 °C, which is 0.0592(h/n) V/pH, where h and n are the number of protons and electrons involved in the electrode process [41]. For analytical purposes, the medium chosen to carry out further studies was the 0.05 mol/L acetate buffer solution at pH 4.0.

### 3.3. Effect of accumulation time

**Figure 6.**
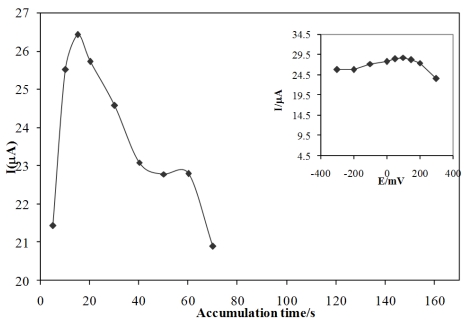
Effect of the accumulation time on the peak current in the presence for diclofenac solution a) 1.0 × 10^−8^ M and at inst figure: 5.0 × 10^−7^ M at acetate buffer pH = 4. E_acc_. = 0.1 V., ƒ = 600 Hz., amplitude□ = 50 mV.

As expected, the extent of pre-concentration is a function of the accumulation time (t_acc_). The dependence of peak current on accumulation time was studied at a diclofenac concentration of 5.0 × 10^−7^ mol/L (Figure 6). The peak current increased with increasing accumulation time up to 20 s and after that it decreased indicating the saturation of the carbon paste electrode by drug causing fouling of the electrode. Hence, an accumulation time of 20 s were chosen to evaluate the best work conditions to the proposed method.

### 3.4. Effect of accumulation potential

The dependence of the peak current on the accumulation potential was evaluated over the range of −0.3 to 0.3 V for 5.0 × 10^−7^ mol/L of diclofenac at 0.05 mol/L acetate buffer pH 4.0 in the presence of the drug, for an accumulation period of 20 s. (See the inset in Figure 6) The results obtained shown that the Ip values are maximum for an accumulation potential of 0.1 V and after that the current decreases so a 100 mV potential was selected as a best accumulation potential.

### 3.5. Optimization of FFT-SW frequency and amplitude

**Figure 7.**
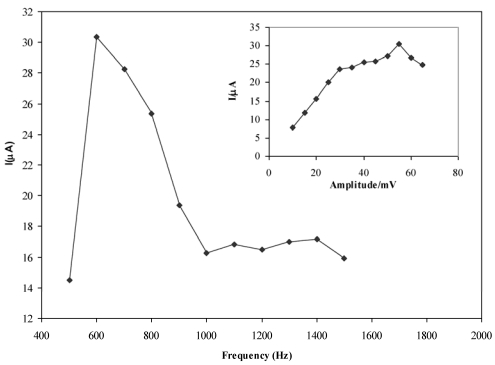
The effect of frequency and amplitude (the inset figure) on the response of CPE for 5.0 × 10^−7^ M diclofenac in 0.05 M of acetate buffer.

To study the effect of these factors, the SW frequency and amplitude between 500–2,000 Hz and amplitude of 5 to 50 mV were examined. In Figure 7 the importance of frequency and amplitude for solutions of diclofenac is demonstrated. In fast voltammetric analysis, the SW frequency and amplitude are important factors since analyte signal, background noise, and peak shape rely on the speed of the excitation signal. It should be noted that the solution resistance, electrode diameter, and stray capacitance of the system will limit the sensitivity gains obtained by raising the SW frequency. However, increasing the SW frequency will increase the SW peak current, or the sensitivity, but this will be tempered by a higher charging/faradic current ratio. Due to this fact, the SW frequency acts like the sweep rate in cyclic voltammetry. Therefore, using very high SW frequencies leads to shorter potential scan times, and consequently, the response peak for the analysis becomes smaller and skewed, due to insufficient time for oxidation of the electrode surface, but for the redox reaction it should be optimized for electron transfer rate. A series of SW frequencies were examined to determine the optimal frequency for the detection of diclofenac. A plot of SW frequency versus ΔQ showed that a frequency of 500 Hz was the instrumental limit for this system. Above this frequency, excessive charging currents interfered with the measurement of the faradic current, decreasing the diclofenac ΔQ. Thus, further studies of SWV detection used 600 Hz with a dc ramp time of 100 ms to provide an overall sampling rate of 20 Hz.

Theoretically, the optimal square wave amplitude for a reversible system is 50/n mV. To determine the influence of SW amplitude on ΔQ, various amplitudes were investigated. Figure 6 shows the effect of SW amplitude on increasing ΔQ that was observed by increasing the amplitude to 50 mV, after which ΔQ began to decrease when amplitudes greater than 50 mV were used. Low frequency noise (baseline drifting) was more pronounced when SW amplitudes above 10 mV were used. SW amplitude of 50 mV and frequency of 600 Hz were thus found to be optimal.

### 3.6. Calibration curves

**Figure 8.**
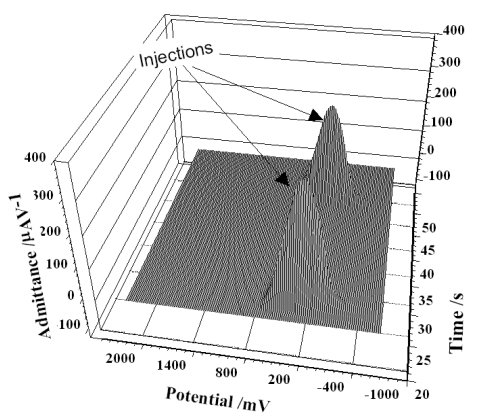
FFT Square-Wave voltammograms at a DyNW/CPE recorded during some injection experiment. The bulk solution was 0.05 M of acetate buffer., and the frequency was 600 Hz. The injected amount of solution contained 1.0 × 10^−6^ M diclofenac.

Figure 8 show the 3-dimensional figure of redox behavior of diclofenac in a flow injection system on the DyN/WCPE in 0.05 M acetate buffer, caused by the addition of a solution of 50 μL of 5.0 × 10^−6^ M diclofenac which was recorded by FFT-SW method. The FFT-SW modulation had amplitude of 50 mV and a frequency of 600 Hz. Before each scan, the electrode was held at E_c1_ potential (1,400 mV) for 60 ms, the E_c2_ potential at −200 mV for 60 ms.

**Figure 9.**
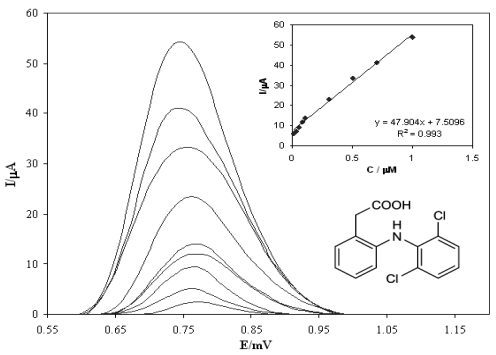
Adsorptive square wave voltammograms obtained for the increasing concentration of diclofenac in acetate buffer (pH = 4) (a-k) at concentration (1, 0.7, 0.5, 0.3, 0.1, 0.08, 0.05, 0.03, 0.02 and 0.01 μM) respectively. Intercept: dependence of peak current on the diclofenac concentration under the optimized conditions mentioned before.

Figure 9 illustrates the obtained calibration carves and SW voltammograms for measurements of Diclofenac in 0.05 M acetate buffer. The experimental conditions were set at optimum values in order to obtain the best detection limits. As mentioned above the electrode response could be expressed in various ways such as peak heights or peak areas. For this reason, the magnitude of injection peaks depends on the choice of the data processing methods. Like stripping voltammetry methods, here, the electrode response is proportional to the electrode coverage [35-39,46,47]. Measurements carried out for small analyte concentrations allow the estimation of the detection limit C_DL_:

$$C_{DL}=\frac{3s_{b}}{\mathrm{slope}}$$

where *sb* is the standard deviation (or noise) of the baseline around the peak.

The linearity was evaluated by linear regression analysis, which was calculated by the least squares regression method [49]. The calibration curves constructed for diclofenac were linear over the concentration range of 1.0–0.01 μM. Peak areas of diclofenac were plotted versus its concentration and linear regression analysis performed on the resultant curve. A correlation coefficient of R = 0.9964 with %R.S.D. values ranging from 0.21%–2.5% across the concentration range studied were obtained from this analysis. Typically, the regression equation for the calibration curve was found to be Y = 47.904 + 7.5096 (R^2^ = 0.9930) Figure 9. shows the calibration graph that was obtained for the monitoring of diclofenac in a 0.05 M acetate buffer. The C_DL_ was measured as the lowest amount of the analyte that may be detected to produce a response which is significantly different from that of a blank. The limit of detection was confirmed by calculations based on the standard deviation of the response (*δ*) and the slope (*S*) of the calibration curve at the levels approaching the limits according to equation C_DL_ = 3.3 (*δ/S*) [49]. The C_DL_ for diclofenac was 2.0 × 10^−9^ M. The LOQ was measured as the lowest amount of analyte that can be reproducibly quantified above the baseline noise, for which triplicated injections resulted in a RSD ≤ 1.7%. A practical LOQ giving a good precision and acceptable accuracy was found to be 5.0 × 10^−9^ M.

### 3.7. Assay of tablets

The method developed in the present study was applied for the determination of diclofenac in tablets (Sobhan Co., Iran, 75 mg) from the Iranian market. The results showed a percent recovery of 99.96% and a R.S.D. of 1.20%.

### 3.8. Analytical applications

After the application of the method to the Iranian market injection, the resulting data showed a recovery percentage value of 99.88 % and a respective R.S.D. value of 1.92 %. The proposed method was also applied to the determination of diclofenac in spiked urine and plasma samples. The results of analysis of spiked human plasma (*n* = 5) and urine (*n* = 5) is shown in Table 1. The results are satisfactory, accurate and precise. No interference was noticed from the urine content after just dilution with the supporting electrolyte. The major advantage of the method as applied to plasma and urine is that no prior extraction step is required.

Data obtained from five replicates at each concentration. Interpolated concentration data expressed as mean ±S.D.

Comparison of the detection limit of the proposed method with the other reported methods is presented in Table 2. It is immediately obvious that the sensitivity of the method is superior to all previously reported methods. The data reveals that the detection limit of the method is about 10 times lower than the most sensitive reported method in Table 2.

## 4. Conclusions

This report described a novel, sensitive, and widely applicable FFT-SWV detection method using a new DyNW/CPE. Like other nanomaterials such as carbon nanotubes this modification with metal oxide nanowires helped improve the sensitivity of the drug determination by reducing the over-potential and increasing the current. It is observed that DyNW could facilitate the exchange of electrons with the electrode. Here FFT-SWV was demonstrated to provide sensitive detection of a drug and it is able to determine a wide range of analytes based on oxidation on the electrode surface. Square-wave adsorptive voltammetry on a carbon paste electrode can be used to determine diclofenac at trace levels because of its low detection limit. Diclofenac can be effectively accumulated from aqueous solutions or urine samples onto the surface of DyNWCPE, increasing the sensitivity of the method. Electrochemical methods for pharmaceutical and biological sample analysis have been proven to be fast, precise, and simple to perform and produce low cost results, in which the interference from excipients of the drugs and interference of the biological fluids do not interfere with the determination, and, consequently, extraction procedures are not needed. The above methods can be suggested as a good alternative for the routine quality control of this antibiotic drug in pharmaceutical formulations.

Finally, the principal advantage of the electroanalytical method is that it was simple, fast and more sensitive than the reported method [7-21] with equivalent precision and accuracy. It is hoped that this will make FFT-SWV easier to use as well as provide enhanced analytical sensitivity. Also, application of FFT-SWV to high-performance liquid chromatography is being considered.

## Figures and Tables

**Figure 1.**
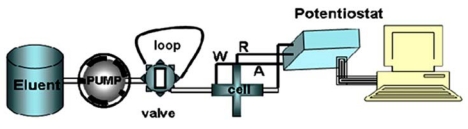
The schematic of the flow injection analysis.

**Table 1.** Application of the proposed method to the determination of diclofenac in spiked humane plasma and urine.

**Added (ng/mL)**	**Interpolated concentration**	**R.S.D (%)**	**R.E. (%)**
5 (plasma)	5.1 ± 0.4	1.2	1.1
5(urine)	4.8 ± 0.1	1.15	0.98

**Table 2.** Application of the proposed method to the determination of diclofenac in spiked humane plasma and urine.

**Method**	**Detection limit**	**Ref. No.**
Potentiometry	3 × 10^−6^ M	7
HPLC-MS	0.5 ng mL^−1^	12
Spectrophotometry	0.37 μg/mL	21
GC	100 pg/mL	18
FFTSWV	2.0 × 10^−9^ M	This work
